# External validation of precisebreast, a digital prognostic test for predicting breast cancer recurrence, in an early-stage cohort from the Netherlands

**DOI:** 10.1186/s13058-025-02104-8

**Published:** 2025-08-20

**Authors:** Pieter J. Westenend, Claudia Meurs, Gerardo Fernandez, Marcel Prastawa, Abishek Sainath Madduri, Aaron Feliz, Juan Carlos Mejias, Alexander Shtabsky, Xiaozhu Zhang, Brandon Veremis, Rebecca DeAngel, Michael J. Donovan

**Affiliations:** 1 Laboratory of Pathology, Dordrecht, Netherlands; 2 PreciseDx, Inc., New York, NY USA; 3 Mount Sinai Health System, New York, NY USA

**Keywords:** Early-stage breast cancer, Digital pathology, Artificial intelligence, Risk stratification, Prognostic model, Recurrence prediction, PreciseBreast

## Abstract

**Background:**

Current clinical guidelines recommend gene expression profiling to guide treatment in early-stage breast cancer. PreciseBreast (PDxBR) is a digital prognostic tool that integrates artificial intelligence (AI)-derived features from hematoxylin and eosin (H&E) slides with clinicopathologic data to predict recurrence risk. This study externally validated PDxBR in an independent cohort and compared its performance to other risk models.

**Methods:**

We retrospectively analyzed PDxBR in a cohort of 739 patients with early-stage hormone receptor-positive, HER2-negative breast cancer (median follow-up of 8.8 years). For each case, one H&E-stained slide was digitized and analyzed to generate recurrence risk scores using the full PDxBR model, as well as image-only and clinical-only variants. A subset of patients who underwent MammaPrint testing was also evaluated. Model performance was assessed by AUC/C-index, hazard ratios, sensitivity, specificity, and negative and positive predictive values (NPV and PPV, respectively).

**Results:**

PDxBR showed prognostic accuracy in this external cohort (AUC/C-index 0.71, 95% CI: 0.66–0.75). Applying the PDxBR threshold (≥ 58 versus < 58) yielded a hazard ratio of 3.05 (95% CI: 2.1–4.4, *p* < 0.001), sensitivity 0.70, specificity 0.59, NPV 0.90, and PPV 0.27. PDxBR outperformed the modified Adjuvant! Online clinical model (MINDACT model, *p* < 0.00001) and effectively reclassified grade 2 tumors into distinct risk groups.

**Conclusions:**

PDxBR demonstrated robust prognostic performance in an independent cohort, supporting its potential as a scalable, reproducible alternative to genomic assays for individualized risk stratification in early-stage breast cancer.

**Supplementary Information:**

The online version contains supplementary material available at 10.1186/s13058-025-02104-8.

## Background

Current clinical practice guidelines from the American Society of Clinical Oncology (ASCO), National Comprehensive Cancer Network (NCCN), and European Society for Medical Oncology (ESMO) recommend comprehensive pathologic assessment of all excised invasive breast cancers, including gene expression profiling when relevant to therapeutic decision-making [1, 2, 3]. Although the global incidence of breast cancer has risen over the past two decades, with an estimated 2.3 million new cases and 670,000 breast cancer-related deaths in 2022 alone, survival rates have improved due to earlier detection and treatment advances [4, 5, 6]. Nevertheless, 10–30% of women with early-stage, estrogen receptor-positive (ER+) and human epidermal growth factor receptor 2-negative (HER2-) breast cancer will experience locoregional or distant recurrence, with approximately half of these events arising more than 5 years after the initial diagnosis [7]. Notably, these recurrence patterns align with reported rates of hormonal therapy resistance, which affect 30–50% of women with early-stage breast cancer [8].

In both the European Union (EU) and the United States (US), 60–70% of patients with early-stage breast cancer are hormone receptor-positive (HR+) and HER2-. These patients face a persistent risk of recurrence that can extend up to 20 years following diagnosis and treatment [7], highlighting the need for accurate and individualized prognostic tools. Inaccurate risk estimation can result in overtreatment, leading to unnecessary systemic therapy exposure and associated toxicities, or undertreatment, potentially leading to preventable recurrence [9, 10].

To address this challenge, a range of prognostic tools has been developed. Genomic assays such as MammaPrint and Oncotype DX integrate genomic, pathologic, and/or clinical data—such as patient age, tumor size, stage, grade, and lymph node status—to enhance individualized recurrence risk prediction and guide treatment decision-making [11, 12, 13]. Despite their value, these assays can be costly, tissue-intensive, and subject to logistical limitations, motivating the search for alternative methods that are more scalable and broadly accessible [14, 15, 16, 17].

In this context, artificial intelligence (AI) and digital pathology have emerged as promising tools for extracting prognostic information from routinely collected hematoxylin and eosin (H&E)-stained slides. These approaches can provide more objective, reproducible assessments of tumor morphology and microenvironmental features that may influence disease progression.

One such application is PreciseBreast (PDxBR), a digital prognostic test that combines routinely available clinicopathologic variables (e.g., age, tumor size, stage and lymph node status) with AI-derived features extracted from whole-slide H&E images of invasive breast cancer. The model generates a continuous risk score (0-100), which stratifies patients into low- and high-risk groups. In its initial development and validation study, PDxBR achieved a concordance index (C-index) of 0.75 and stratified patients between risk groups with a hazard ratio of 4.4 [16, 17]. Thus, PDxBR not only standardizes histologic grading—which is notoriously prone to interobserver variability—but also enhances prognostic accuracy in a reproducible and cost-effective way [18]. 

The current study aimed to externally validate PDxBR in an independent cohort of patients with early-stage HR + breast cancer from the Netherlands (NTH), managed according to ESMO and Dutch clinical guidelines. We also evaluated the performance of image-only (AI-grade) and clinical-only variants of the PDxBR model and compared these models to MammaPrint-based risk stratification in a subset of patients who underwent MammaPrint testing as part of routine care. This approach allowed us to assess the reproducibility of PDxBR prognostic performance over a median follow-up of 8.8 years, and its clinical relevance in comparison to existing genomic tools—mirroring the subgroup analysis in the original validation study with the 21-gene assay.

## Methods

### Study design and participants

We conducted a retrospective longitudinal validation study of the PDxBR prognostic model using an independent cohort of patients with early-stage breast cancer and a median follow-up of 8.8 years. The study cohort was identified by the site principal investigator (P.W.) at the Laboratory of Pathology, Albert Schweitzer Hospital, Dordrecht, NTH. Eligible patients were ≥ 23 years of age and had a diagnosis of infiltrating ductal carcinoma or mixed ductal and lobular carcinoma of the breast. Patients with a history of neoadjuvant therapy or prior breast cancer were excluded. Outcome data were obtained from electronic medical records at Albert Schweitzer Hospital and treatment data were accessed from the Dutch National Cancer Registry. A subset of high risk patients according to Adjuvant! Online (performed at time of diagnosis) were offered the option to have MammaPrint gene expression testing. Given the potential influence of this test on treatment decisions and outcomes, this subgroup was evaluated both as part of the total cohort and separately.

Each patient was assigned a unique study identifier (ID), which was used to link digitized histology slides to the corresponding clinical data. H&E-stained slides were scanned at 40X magnification using a Philips UltraFast slide scanner at the Philips facility in Eindhoven, NTH. The resulting digital images, labeled with study IDs, were then securely transferred to PreciseDx (New York, NY) via encrypted portable hard drives. All images were independently reviewed by three pathologists (A.S., B.V., and G.F.) to confirm the diagnosis of invasive breast cancer and to exclude tumors with pure lobular, mucinous, papillary, or metaplastic histology. These pathologists also verified tumor adequacy and image quality (minimum tumor diameter of > 1 mm). All personnel involved in image review and analysis were blinded to patient outcomes.

For each case, one representative image was selected for digital feature extraction using the PreciseDx digital analysis platform. The technical details regarding the development and validation of the PDxBR digital morphometric features and recurrence risk score have been previously published [16, 17]. Briefly, digital biomarker features were derived to represent protein morphometric correlates of the Nottingham Grading System (NGS) for invasive breast cancer, including measures of tumor architecture, mitotic activity, and lymphocytic or mononuclear infiltrates [19, 20]. These features were integrated with clinical variables—including patient age, tumor size, anatomic stage, and lymph node status—using a validated algorithm to generate a PDxBR recurrence risk score, which classifies patients as low-risk (score of < 58) or high-risk (score of ≥ 58) on a 0-100 scale. To compare the relative individual contribution of digital histology and clinical data, we also evaluated two additional models: an AI-grade image-only model (excluding clinical data) and a clinical-features-only model.

De-identified clinical data—including patient demographics, clinicopathologic features, MammaPrint scores, adjuvant treatment, and outcomes—were abstracted by the NTH study investigator and independently reviewed by PreciseDx personnel for completeness and adjudication, in collaboration with staff located in NTH. Menopausal status was not available and was inferred based on age, with patients ≤ 50 years classified as premenopausal and those > 50 years as postmenopausal.

This study was approved for academic research use and was conducted under a data use agreement between Stichting Albert Schweitzer Ziekenhuis and PreciseDx, in accordance with the General Data Protection Regulation and applicable local data protection laws.

### Statistical analyses

Performance of the PDxBr model was evaluated using the area under the curve (AUC)/C-index, Kaplan-Meier (KM) survival analysis, associated metrics and confidence intervals (CI), including negative predictive value (NPV), positive predictive value (PPV), hazard ratios, sensitivity, and specificity. The PDxBR model was compared to three alternate models: an AI-grade image-only model (PDxBR AI-grade), a PDxBR clinical model (PDxBR Clinical), and a clinical model based on a modified Adjuvant! Online in the MINDACT trial, (referred to as the MINDACT model for this publication), which included ER and HER2 status, histologic grade, and nodal status [21]. The primary endpoint was median 8.8-year invasive disease-free survival, defined as freedom from locoregional recurrence, distant metastasis, and death from any cause. Cause-specific mortality could not be distinguished from overall survival in this cohort.

This study followed the Transparent Reporting of a multivariable prediction model for Individual Prognosis or Diagnosis (TRIPOD) guidelines to ensure comprehensive and transparent reporting of predictive model development and validation.

## Results

### Patient and tumor characteristics

A total of 739 eligible participants with early-stage breast cancer were identified through the Albert Schweitzer Hospital Department of Pathology and the Dutch National Cancer Registry. The median age was 61 years, with 78% of patients over 50 years, and a majority (~ 85%) identified as White. Of the excised tumors, 645 (87%) were ≤ 2.5 cm, 729 (99%) were anatomic stage I or II, 587 (79%) were node-negative (N0), 381 (52%) were histologic grade 2, 739 (100%) were ER+, and 739 (100%) were HER2- (Table 1).

**Table 1:** Demographics of NTH cohort

Characteristic, *n* (%)	*N* = 739
**Age at Diagnosis (years)**, median (SD)	61 (29–91)
Age ≤ 50	160 (21.7)
Age > 50	579 (78.4)
**Tumor Size (cm)**	
≤2.5	645 (87.3)
>2.5 and ≤ 5	86 (11.6)
>5	8 (1.1)
**Anatomic Stage**	
I	454 (61.4)
II	275 (37.2)
III	9 (1.2)
IV	1 (0.1)
**Positive Lymph Nodes**	
0	587 (79.4)
1–3	152 (20.6)
**Tumor Grade** ^**+**^	
1	266 (36.0)
2	381 (51.6)
3	86 (11.6)
**ER Status**	
Positive	739 (100.0)
**PR Status**	
Positive	648 (87.7)
Negative	75 (10.2)
NA	16 (2.2)
**HER2 Status**	
Negative	739 (100.0)
**Prior Treatments**	
Chemotherapy	214 (29.0)
Endocrine Therapy	475 (64.3)
Radiation Therapy	503 (68.1)
**Total Events**	130 (17.6)
Locoregional Recurrences/Second Primaries	24 (3.3)
Distant Metastases	25 (3.4)
Deaths	81 (11.0)
**Risk Group Stratification**	
***Prior MammaPrint Analysis*** *	
Low-risk	166 (65.9)
High-risk	86 (34.1)
Unknown	
***PDxBR***	
Low-risk	400 (54.1)
High-risk	339 (45.9)
***PDxBR Clinical***	
Low-risk	479 (64.8)
High-risk	260 (35.2)
***PDxBR AI-grade***	
Low-risk	372 (50.3)
High-risk	367 (49.7)
**Follow-Up Time (months)**, median (range)	105.5 (2.4-160.7)

*n = 252 patients with MammaPrint Results; ^+^, histologic grade was not available for 6 patients

AI, artificial intelligence; cm, centimeters; ER, estrogen receptor; HER2, human epidermal growth factor receptor 2; NA, not available; PDxBR, PreciseBreast; PR, progesterone receptor

Prior treatments included chemotherapy in 214 (29%) patients, endocrine therapy in 475 (64%), and radiation therapy in 503 (68%) (Table 1). The absence of endocrine therapy in 36% of the overall cohort was not unexpected, as Dutch guidelines differ from ESMO recommendations. Approximately 70% of this group had radiation and/or chemotherapy. Specifically, endocrine therapy is not advised for patients over 34 years with N0/N0(i+) disease and grade 1 tumors up to 2 cm or grade 2–3 tumors up to 1 cm, or for patients under 35 years of age with N0/N0(i+) disease and grade 1 tumors up to 1 cm.

Among the 739 patients, there were 130 (18%) recurrence or survival events over the median follow-up time of 8.8 years: 24 (3.3%) locoregional recurrences or second primaries, 25 (3.4%) distant metastases, and 81 (11.0%) deaths (Table 1). Among the 25 patients with distant metastases, 76% were > 50 years, 68% had tumors < 2.5 cm, 72% were N0, 92% were stage I or II, 60% were histological grade 2, 100% received endocrine therapy, and 60% received chemotherapy (data not shown). Of the 739 total patients, 252 (34.1%) received MammaPrint testing, of which 166 (65.9%) were classified as low-risk and 86 (34.1%) were classified as high-risk (Table 1).

### Performance of PreciseBreast

The PDxBR model identifies the number of positive lymph nodes and patient age at diagnosis as the most predictive clinical variables for breast cancer recurrence, while tubule formation (glandular architecture) and tumor-infiltrating lymphocytes are the most influential image-derived features [17]. A SHapley Additive exPlanations (SHAP) plot detailing the differential weighting and contribution of individual morphological features of the AI-grade model output is presented in Supplemental Figure S1. Of importance, gland differentiation has a 29.1% absolute contribution to the PDxBR score (encompassing tubule formation [20.7%], neighboring nuclei [4.4%], and sheets [4.0%]), while nuclear pleomorphism represents 13.7% and mitotic figure count is at 8.2%. Collectively, the imaging features accounted for 51% of the PreciseBreast score. A univariate Cox proportional hazards model and C-index analysis identified gland architecture as one of the most informative components of the PDxBR model along with the independent contribution of nuclear pleomorphism and mitotic figures (Supplemental Table S1). Notably, although both HR status and histologic grade were among the offered variables during model development, they were excluded during feature selection, suggesting that their prognostic value was subsumed by the AI-derived morphologic features.

**Fig. 1:**
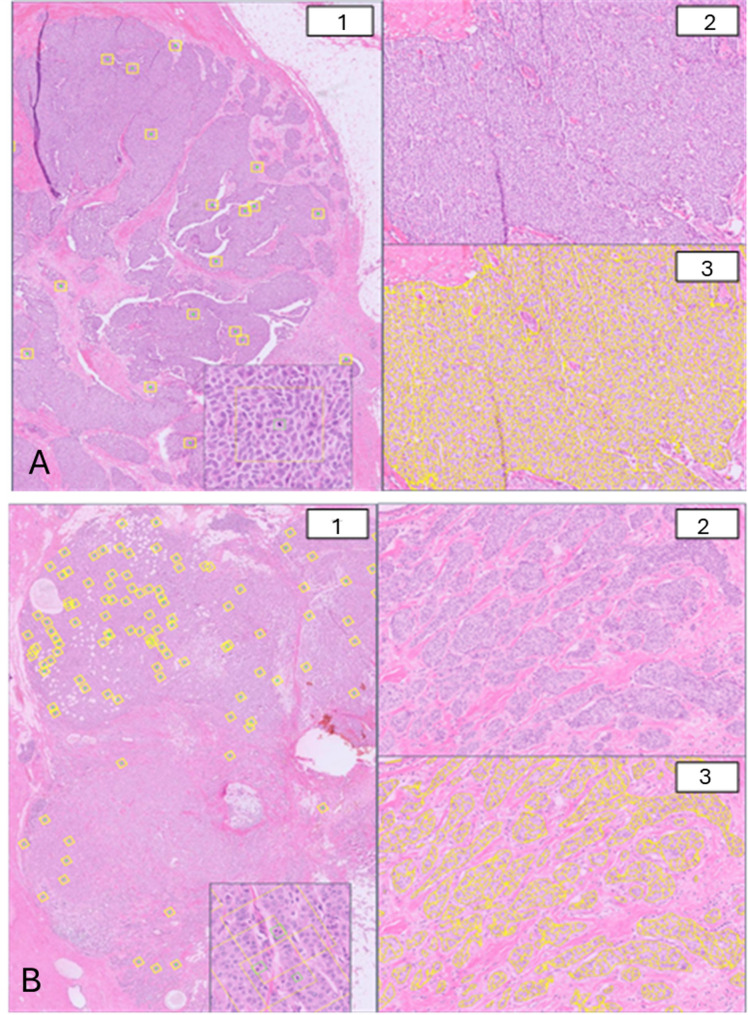
Representative H&E-stained sections of patients misclassified as low-risk by MammaPrint and the MINDACT model but correctly stratified as high-risk by PreciseBreast. (**A**) Patient 1, aged 62 with stage I/II invasive breast cancer (tumor size 1.2 cm, grade 2, N0) who developed a distant metastasis. PreciseBreast score was 67.8 and classified as high-risk by the PDxBR AI-grade model. (**B**) Patient 2, aged 55 with stage I/II invasive breast cancer (tumor size 1.1 cm, grade 3, N0) and a locoregional recurrence. Her PreciseBreast borderline low-risk score of 56 (risk threshold: 58) and was classified as high-risk by the PDxBR AI-grade model. Both patients had ER+/PR+/HER2- tumors. For each case, panel 1 shows mitotic figure detection (yellow boxes with green inset boxes), and panel 2 shows an H&E section that in panel 3 displays gland architecture overlay used to assess tubule formation

The clinical relevance of these AI-derived features is illustrated by two cases that were misclassified as low-risk by both MammaPrint and the MINDACT model but correctly identified as high-risk by the PDxBR model (Fig. 1). In both cases, patients with ER+/HER2- tumors experienced recurrence—one distant and one locoregional—despite being assigned to low-risk groups by traditional tools. These examples underscore the ability of PDxBR to identify high-risk patients through AI-derived morphologic features that may be overlooked by existing clinical and genomic models.

Building on these insights, we assessed the overall performance of PDxBR and its component models in the full NTH cohort (*n* = 739). PDxBR demonstrated an AUC/C-index of 0.71 (95% CI: 0.66–0.75) (Table 2), compared to the original validation study which had an AUC/C-index of 0.75 (95% CI: 0.72–0.79) [17]. The PDxBR AI-grade model and clinical model yielded AUC/C-indices of 0.66 (95% CI: 0.62–0.71) and 0.69 (95% CI: 0.65–0.74), respectively. The MINDACT model [21], which incorporates ER status, HER2 status, histological grade, tumor size, and nodal status, had an AUC/C-index of 0.60 (95% CI: 0.56–0.64). We further evaluated the performance of the PDxBR model to the PDxBRAI-grade and PDxBR clinical models using DeLong’s test for paired C-index comparison. The PDxBR model significantly outperformed PDxBR AI-grade (*p* < 0.0001), while the PDxBR versus PDxBR clinical showed no statistical difference (*p* = 0.49). Furthermore, performance of the PDxBR clinical versus PDxBR AI-grade model was not statistically different (*p* = 0.076), although trending towards significance. Collectively, these results support prognostic superiority when both clinical and imaging features are algorithmically combined to risk stratify patients.

**Table 2:** Multi-model risk performance in the NTH cohort

Test	C-Index (95% CI)	Hazard Ratio (95% CI)	SE (95% CI)	SP (95% CI)	PPV (95% CI)	NPV (95% CI)
**MammaPrint**	0.52 (0.39–0.65)	1.19 (0.43–3.29)	0.38 (0.18–0.62)	0.66 (0.6–0.72)	0.07 (0.03–0.15)	0.94 (0.89–0.97)
**PDxBR**	0.71 (0.66–0.75)	3.05 (2.10–4.44)	0.70 (0.62–0.77)	0.59 (0.55–0.63)	0.27 (0.22–0.32)	0.90 (0.87–0.93)
**PDxBR AI-grade**	0.66 (0.62–0.71)	2.87 (1.96–4.19)	0.71 (0.62–0.78)	0.55 (0.51–0.59)	0.25 (0.21–0.30)	0.90 (0.86–0.92)
**PDxBR Clinical**	0.69 (0.65–0.74)	2.98 (2.10–4.24)	0.60 (0.51–0.68)	0.70 (0.66–0.74)	0.30 (0.25–0.36)	0.89 (0.86–0.92)
**MINDACT** **Model**	0.60 (0.56–0.64)	2.17 (1.54–3.06)	0.52 (0.44–0.61)	0.68 (0.64–0.72)	0.26 (0.21–0.31)	0.87 (0.84–0.90)

AI, artificial intelligence; CI, confidence interval; NPV, negative predictive value; NTH, The Netherlands; PDxBR, PreciseBreast; PPV, positive predictive value; SE, sensitivity; SP, specificity

**Fig. 2:**
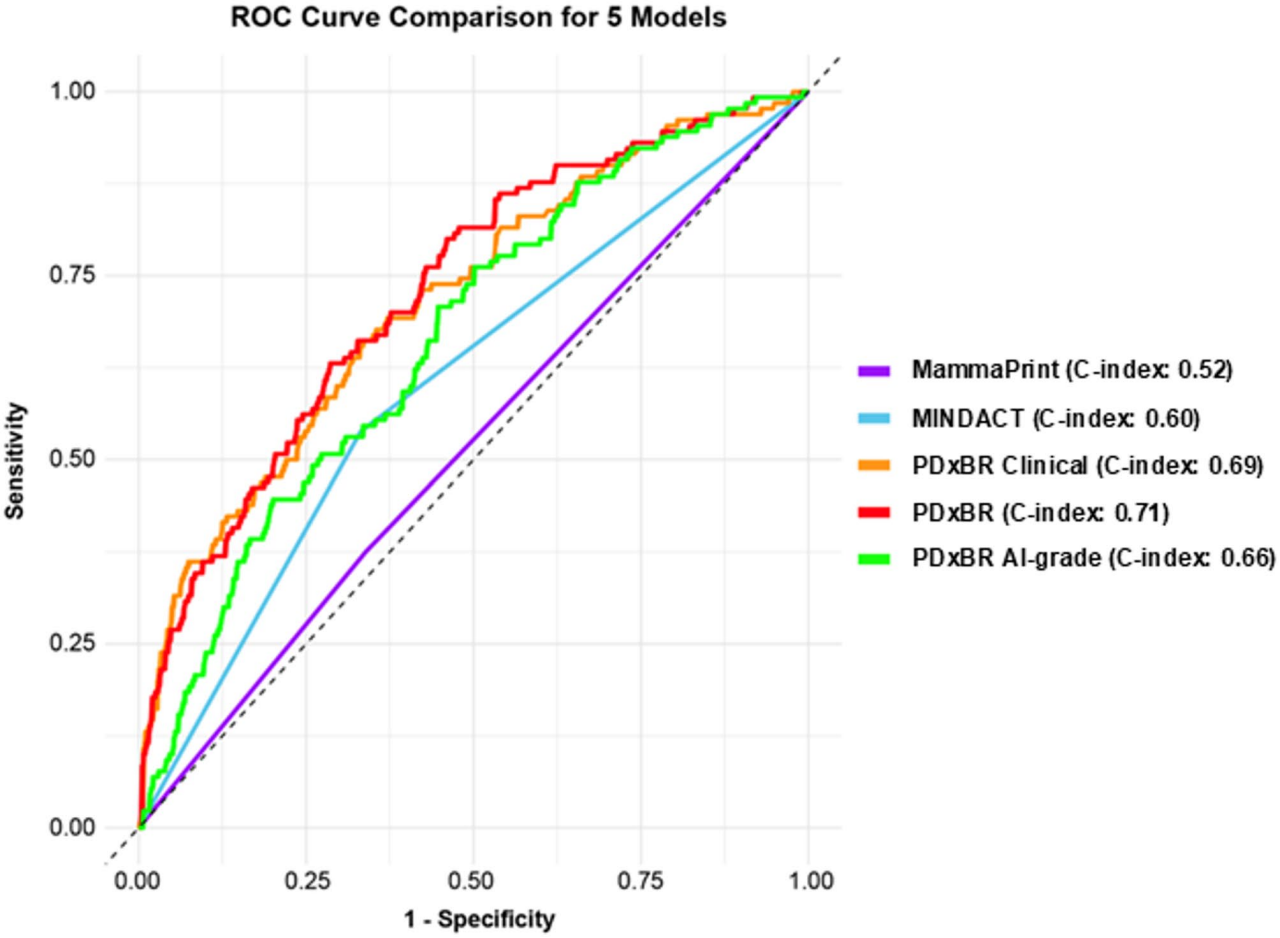
AUC comparison of model performance for predicting breast cancer recurrence within a 6-year period. The DeLong test was used to assess statistical differences between AUCs: PDxBR (C-index = 0.71), PDxBR Clinical (0.69), PDxBR AI-grade (0.66), MINDACT (0.60), and MammaPrint (0.52). PDxBR significantly outperformed MammaPrint (*n* = 252, *p* = 0.0004) and the MINDACT model (*p* < 0.00001). The PDxBR Clinical model also significantly outperformed MINDACT (*p* < 0.00001), further supporting its enhanced prognostic accuracy.

An exploratory model incorporating only histologic grade and tumor size demonstrated an AUC/C-index of 0.60 (95% CI: 0.56-0.64), identical to that of MINDACT (data not shown). An AUC comparison of all models demonstrated that PDxBR significantly outperformed the MINDACT model (DeLong test *p* < 0.00001 (Fig. 2).

Using a risk score cutoff of 58 (< 58 = low-risk, ≥ 58 = high-risk), PDxBR stratified patients with a hazard ratio of 3.05 (95% CI: 2.1–4.4, *p* < 0.001), sensitivity of 0.70, specificity of 0.59, NPV of 0.90, and PPV of 0.27 for predicting breast cancer recurrence (Table 2). These results were consistent with those observed in the original validation study, which reported a hazard ratio of 4.4 (95% CI: 2.7–7.1, *p* < 0.001), sensitivity of 0.61, specificity of 0.77, NPV of 0.94, and PPV of 0.24 [17].

**Fig. 3:**
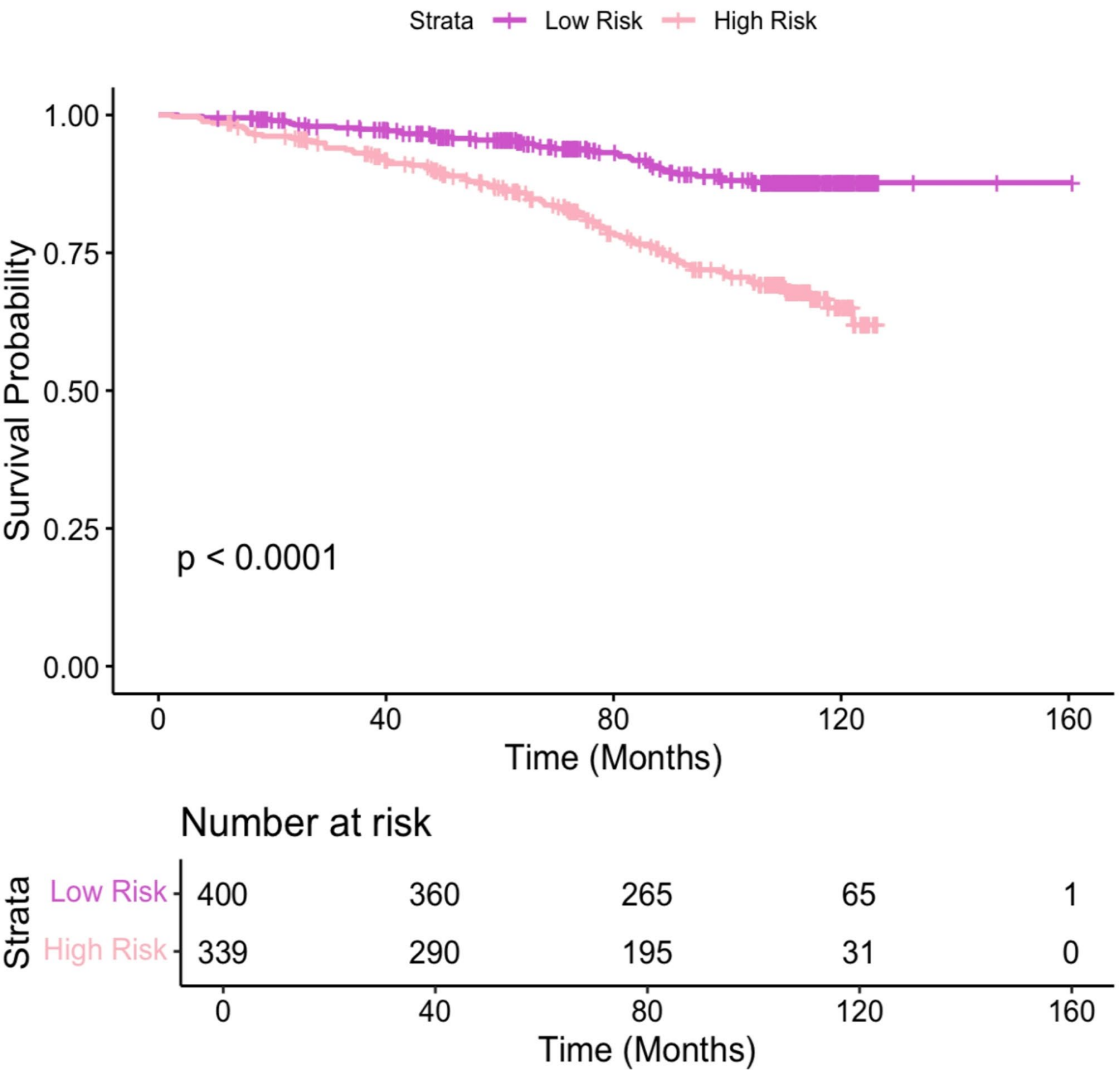
Kaplan-Meier analysis of invasive disease-free survival stratified by PDxBR risk score in the NTH Cohort. Patients classified as high-risk (*n* = 339) had significantly worse predicted invasive disease-free survival compared to those classified at low-risk (*n* = 400) over a median follow-up of 8.8 years (*p* < 0.0001). Risk tables show the number of patients at risk over time in each group.

In the NTH cohort, PDxBR classified 400 (54%) patients as low-risk and 339 (46%) as high-risk (Table 1). KM survival analysis of the NTH cohort demonstrated a significant difference in predicted invasive disease-free survival between PDxBR-defined risk groups, with high-risk patients displaying a significantly lower survival probability compared to those classified as low-risk (Fig. 3, *p* < 0.0001).

The NTH cohort included 130 recurrence events, of which 91 (70%) were classified as high-risk by PDxBR (including 61 deaths, 16 metastases, and 14 locoregional or second primary recurrences), compared to 68 (52%) events classified as high-risk by the MINDACT model. Detailed model performance metrics and event types are provided in Table 2 and Supplemental Table S2, respectively. Across the entire 739 patient cohort, 125 patients who experienced an event were classified as low-risk according to the MINDACT model, but high-risk by PDxBR. In contrast, only 34 patients with an event were classified as high-risk by MINDACT model and low-risk by PDxBR. Thus, overall PDxBR exhibited good risk stratification and event discrimination when compared to the MINDACT model.

By comparison, the original validation cohort had 69 recurrence events, with 39 (57%) classified as high risk [17]. Moreover, the longer median follow-up period in the NTH cohort (8.8 years versus 6 years in the original validation study), along with higher event counts—81 versus 32 deaths, 25 versus 20 distant metastases, and 24 versuss 17 locoregional or second primary recurrences (Table 1) provides valuable contextual insight.

We also evaluated PDxBR performance specifically in patients who developed distant metastases (*n* = 25). Among these, the majority of metastatic events in PDxBR high-risk patients occurred after 5 years of follow-up (Supplemental Figure S2).

### AI-grade and clinical model performance

**Fig. 4:**
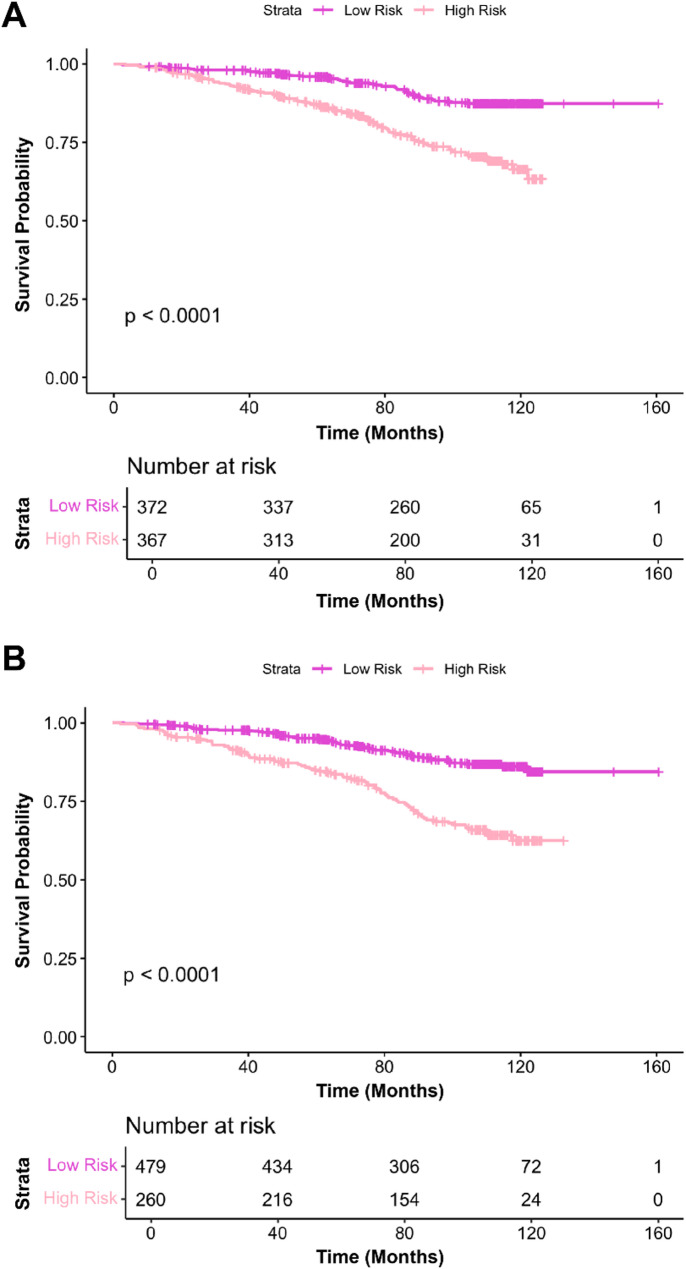
Kaplan-Meier analysis of invasive disease-free survival stratified by PDxBR AI-grade and Clinical model risk scores in the NTH cohort. (**A**) In the PDxBR AI-grade image-only model, patients classified as high-risk (*n* = 367) had significantly worse predicted invasive disease-free survival compared to those classified as low-risk (*n* = 372) over a median follow-up of 8.8 years (*p* < 0.0001). (**B**) In the PDxBR Clinical model, patients classified as high-risk (*n* = 260) had significantly worse predicted invasive disease-free survival compared to those classified as low-risk (*n* = 479) over a median follow-up of 8.8 years (*p* < 0.0001). Risk tables show the number of patients at risk over time in each group.

The PDxBR AI-grade and clinical models yielded hazard ratios of 2.87 (95% CI: 1.96–4.19) and 2.98 (95% CI: 2.1–4.24), respectively (Table 2). Both models demonstrated similar NPVs of 0.90 and 0.89, and PPVs of 0.25 and 0.30, respectively. Notably, the AI-grade high-risk model identified 92 (71% of all 130 events), including 21 (84%) metastases and 13 (54%) recurrences (Supplemental Table S2). KM survival analysis of the NTH cohort by both the PDxBR AI-grade (Fig. 4A) and PDxBR Clinical models (Fig. 4B) also demonstrated a significant difference in predicted invasive disease-free survival between risk groups in the NTH cohort, with high-risk patients displaying a significantly worse survival probability compared to those classified as low-risk.

When evaluating performance specifically for predicting distant metastases, the AI-grade model produced a hazard ratio of 6.35 (95% CI: 2.17–18.53) and a sensitivity of 0.84. The NPVs for the PDxBR, PDxBR AI-grade, and PDxBR Clinical models were 0.98, 0.99, and 0.98, respectively (Supplemental Table S3).

As noted in Table 1, only 475 (64%) of all patients received endocrine therapy and 214 (29%) received chemotherapy. Treatment patterns were consistent with clinical risk as defined by MINDACT criteria: 92% (243/263) of high-risk patients received endocrine therapy and 51% (133/263) received chemotherapy. In comparison, among those classified as high-risk via PDxBR, 81% (275/339) received endocrine therapy and 32% (107/339) chemotherapy. Finally, of the 25 patients who developed distant metastases, 18 (72%) received endocrine therapy and 13 (48%) received chemotherapy (data not shown).

### Use of PDxBR and PDxBR AI-grade to redistribute NGS grade

The PDxBR model has previously demonstrated the ability to recategorize patients graded under the NGS three-tier system into binary low- versus high-risk categories. As such, this yielded an AUC analysis over a 5-year period comparing histologic grade versus AI-grade and demonstrated good discrimination with a DeLong *p* < 0.001 (Supplemental Figure S3). The redistribution of histologic grading with the AI-grade model in Supplemental Table S4 further illustrates the ability to provide an objective assessment of grade 1 and 3 tumors while highlighting the higher risk associated with the grade 2 category. Further, the reclassification is particularly relevant for grade 2 tumors, which accounted for 52% (381/739) of the total study population (Table 1). Of these, PDxBR reclassified 175 (46%) as low-risk and 206 (54%) as high-risk. Similarly, 195 of 266 (73%) patients with grade 1 tumors were classified as low-risk and 61 of 86 (71%) patients with grade 3 tumors as high-risk.

**Fig. 5:**
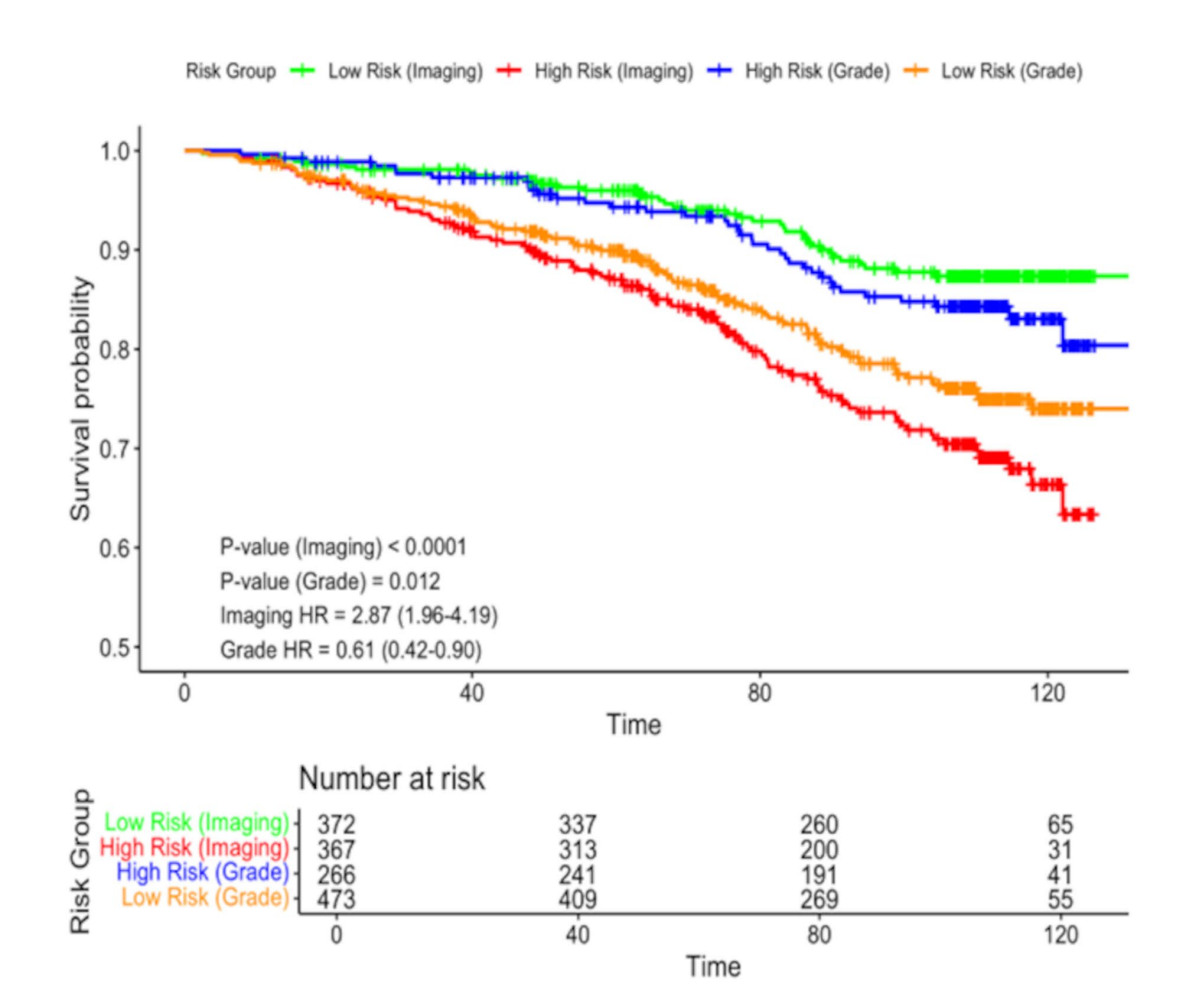
Histologic versus PDxBR AI-grade on the 739 patients in the NTH cohort. Histologic tumor grades 2 and 3 were merged into a single high-risk category (blue line, Grade) vs. low-risk that consisted of grade 1 tumors only (brown line, Grade). This was compared to the PDxBR AI-grade high-risk (red line) and low-risk (green line) categorization, which stratifies risk beyond traditional histologic grading.

When applying the PDxBR AI-grade model, patients with grade 2 tumors were more evenly distributed between risk categories, with the majority of grade 1 and grade 3 tumors classified as low-risk (76%) and high-risk (81%), respectively. Given that grade 2 tumors are generally (clinically) considered higher risk, we arbitrarily combined histologic grades 2 and 3 into a single high-risk category (blue line, Fig. 5) vs. low-risk that consisted of grade 1 tumors (brown line) and compared them to a robust PDxBR AI-grade high-risk (red line) and low-risk (green line) categorization, beyond traditional histologic grading (Fig5). Next, we performed KM survival analyses for low-grade versus low-risk PDxBR AI-grade and high-grade versus high-risk PDxBR AI-grade groups. No statistical difference in survival between either grouping was determined (Supplemental Figure S4A, *p* = 0.38 and Figure S4B, *p* = 0.59, respectively). Lastly, we conducted a KM survival analysis restricted to patients with grade 2 tumors stratified by PDxBR low- and high-risk classification and observed a significant difference in recurrence-free survival between groups (log-rank *p* = 0.00017, HR = 2.74 (95% CI:1.59–4.73), Supplemental Figure S5). In addition, the PDxBR AI-grade model identified 21 of 25 patients with metastatic events, compared to 16 and 17 identified by the PDxBR and MINDACT models, respectively (Supplemental Table S2).

### Evaluation of the MammaPrint subgroup with PreciseBreast

To further assess PDxBR performance, we evaluated a subgroup of 252 patients who had previously undergone MammaPrint testing. In this cohort, MammaPrint was used to both predict risk of distant recurrence and to guide adjuvant treatment decisions. As a result, the outcomes from this analysis may be biased towards the PDxBR risk assessment tool. This group had clinicopathologic characteristics like the overall cohort of 739 patients, with a median age of 57 years (73% aged > 50), 99% with stage I or II disease, 84% N0, 66.7% with grade 2 tumors, and 100% HR+/HER2- status (Supplemental Table S5). There were 16 (6%) events, comprising 4 locoregional recurrences, 5 metastases, and 7 deaths. Median follow-up was 5 years for all patients versus 4 years for the 16 patients with events. Most of these patients were > 50 years of age (88%), 94% had tumors ≤ 2.5 cm, 88% were N0, and 94% had grade 1 tumors.

The AUC/C-index of MammaPrint in this subgroup was 0.52 (95% CI: 0.39–0.65), compared to 0.51 (95% CI: 0.41–0.61) for PDxBR (Table 2 and Supplemental Table S6). Among the NTH cohort with available MammaPrint results, 166 (66%) patients were classified as low-risk and 86 (34%) as high-risk (Table 1). Of the 86 MammaPrint-classified high-risk patients, 70 (81%) received adjuvant chemotherapy and 82 (95%) received endocrine therapy, while among low-risk patients, 5 (3%) received chemotherapy and 134 (81%) received endocrine therapy.

MammaPrint identified 6 of 16 events (3 deaths, 3 metastases) in its high-risk group (Supplemental Table S7), whereas PDxBR identified 5 of 16 events (2 deaths, 2 metastases, 1 recurrence) in its high-risk group (*n* = 107) (Supplemental Table S8). PDxBR AI-grade high-risk identified 10 of 16 events (5 deaths, 3 metastases, 2 recurrences), while PDxBR Clinical high-risk identified 3 events (1 death, 1 metastasis, 1 recurrence).

The hazard ratio for MammaPrint was 1.19, with an NPV of 0.94 and PPV of 0.07 (Supplemental Table S6 and Supplemental Figure S6, *p* = 0.73). In comparison, PDxBR had a hazard ratio of 0.55, NPV of 0.92, and PPV of 0.05. In contrast, the PDxBR AI-grade model had a hazard ratio of 1.28, with an NPV of 0.95 and PPV of 0.07. These results promote the potential for PDxBR AI-grade to provide independent prognostic value in patient risk stratification.

## Discussion

We previously validated the prognostic digital assay, PreciseBreast (PDxBR), for its ability to predict the likelihood of invasive breast cancer recurrence within six years following surgical excision [17]. This approach integrates a novel AI-derived tumor grading system—based on morphological and protein expression features from pathology images—with standard clinicopathological variables (age, tumor size, stage, and lymph node status) to predict invasive disease-free survival, with an AUC of 0.76, NPV of 0.94 and PPV of 0.24 [17]. To expand on these initial findings, we evaluated the performance of PDxBR in an independent cohort of early-stage breast cancer patients from NTH, followed for a median of 8.8 years. Compared to the original validation cohort [17], the NTH population represented a lower-risk population, with no cases of combined HER2 + and triple-negative breast cancer, in contrast to the 20% in the original cohort. Despite this, the NTH cohort experienced a slightly higher event rate (16% versus 14%), possibly due to differences in follow-up duration (8.8 versus 6 years).

PDxBR demonstrated similar predictive performance of recurrence risk across both cohorts. In the NTH dataset, the PDxBR model achieved an AUC/C-index of 0.71 (95% CI: 0.66–0.75), compared to 0.75 (95% CI: 0.72–0.79) in the original validation study [17]. The PDxBR AI-grade model yielded an AUC/C-index of 0.66 (95% CI: 0.62–0.71) and the PDxBR Clinical model an index of 0.69 (95% CI: 0.65–0.74), versus 0.67 (95% CI: 0.63–0.71) and 0.71 (95% CI: 0.66–0.76), respectively, in the original cohort. In contrast, a model based on features used in the MINDACT trial (e.g., HR/HER2 status, histologic grade, tumor size, and nodal status), produced a lower AUC/C-index of 0.60 (95% CI: 0.56–0.64). These results highlight the need for standardized methods for optimized breast cancer prognostication [21].

When NTH patients were stratified using PDxBR risk score (≥ 58 versus < 58), the model yielded a hazard ratio of 3.05 ([95% CI: 2.1–4.4], *p* < 0.001), with a sensitivity of 0.70, specificity of 0.59, NPV of 0.90, and PPV of 0.27 for predicting breast cancer recurrence. The original validation study reported a hazard ratio of 4.4 ([95% CI: 2.7–7.1], *p* < 0.001), with a sensitivity of 0.60, specificity of 0.77, NPV of 0.94, and PPV of 0.24 [17]. As expected, the NPV and PPV varied between cohorts, reflecting differences in population characteristics, event frequency and types, and follow-up duration. For example, the rates of recurrence, metastasis, and death differed between the NTH and original cohorts (recurrence: 3.3% versus 25%; metastasis: 3.4% versus 29%; deaths: 11% versus 46%). Despite these differences, PDxBR successfully identified 91 of 130 (70%) patients with events as high-risk in the NTH cohort, compared to 70 of 130 (54%) patients using the clinical model from the MINDACT trial. These findings further support the robustness of PDxBR in stratifying recurrence risk across distinct patient populations.

The American Joint Committee on Cancer (AJCC) 8th and 9th edition staging manuals now incorporate prognostic staging for breast cancer, where histologic grade remains a factor—alongside estrogen and progesterone receptor status, HER2 amplification, and select molecular assays—in assessing recurrence risk. However, histologic grading remains a subjective process, with strong interobserver agreement only for grade 1 tumors, and moderate to fair agreement for grades 2 and 3 [22, 23]. This poses a particular challenge in the NTH cohort, where grade 2 tumors account for 52% of cases. We previously demonstrated that both the PDxBR and PDxBR AI-grade models could resolve this ambiguity by subdividing grade 2 tumors into biologically meaningful low- and high-risk categories. This was confirmed in the NTH cohort, where the 381 patients with grade 2 disease were reclassified by PDxBR into 175 (46%) low-risk and 206 (54%) high-risk groups, with a similar even split using the AI-grade model. These results suggest that reclassifying grade 2 tumors using the AI-grade model—and stabilizing classification for grades 1 and 3—may provide a more objective and accurate basis for risk stratification, thereby enhancing precision in prognostic assessment.

Clinical risk remains a critical component of individualized breast cancer management, as evidenced by tools like RSClin and RSClin + for Oncotype DX [11, 12, 13], and the EndoPredict 12-gene assay, which integrates molecular data with tumor size and nodal status to generate the EPclin score [24]. In this context, we developed an objective tool that quantifies and standardizes readily available clinicopathologic variables (e.g., age, tumor size, stage, and lymph node status) and integrates these with seven morphological features (AI-grade) associated with biological quiescence, disorder, resistance mechanisms, proliferation, and immune response [16, 25, 26, 27].

The comparable performance of the PDxBR Clinical model and the MINDACT trial algorithm further highlights the potential of AI-driven grading to standardize clinical risk assessment, which has historically relied on heuristic and often subjective measures. Our objective is to improve risk stratification for all patients with early-stage invasive breast cancer by introducing interpretable, scalable methodologies that can be seamlessly integrated into routine clinical care. Other groups are developing assays to predict breast cancer recurrence by interrogation of H&E stained whole slide images of invasive breast cancer (IBC). The approaches vary from applying standard histologic grades to identify and train digital features that improve risk discrimination for intermediate grading [28], to constructing foundational self-supervised machine learning models that collectively assess the IBC phenotype and combine these attributes with clinical data to predict risk of recurrence [29].

While our findings support the robustness and clinical utility of PDxBR, several limitations should be considered when interpreting the results. There were notable differences in outcomes and event distributions between the original validation study and the NTH cohort, particularly the longer median follow-up in the NTH cohort (6 versus 8.8 years), as well as a higher number of deaths (32 versus 80) and metastases (20 versus 25). To address the potential for performance bias, we analyzed all event categories—both individually and collectively—across all models, including the subgroup of 252 patients who were also evaluated with MammaPrint. Moreover, the MammaPrint cohort was not designed to independently predict systemic therapy (by MammaPrint) and assess risk for possible events. Additionally, the study design was unable to determine cause-specific mortality, a constraint shared with the original validation study. Finally, since the NTH does not routinely perform Ki67 staining, we were not able to assess its impact on outcome prediction in this patient cohort.

## Conclusions

In this external validation study, the PDxBR digital prognostic model demonstrated robust performance in stratifying recurrence risk among patients with early-stage HR+/HER2- breast cancer treated according to ESMO and Dutch national guidelines. Despite differences in clinical risk profiles, follow-up duration, and event types compared to the original validation cohort, PDxBR maintained prognostic accuracy and outperformed traditional clinical models, including one based on the MINDACT trial. By integrating clinicopathologic variables with AI-derived histologic features, the PDxBR model offers a reproducible and scalable alternative to genomic assays. Furthermore, PDxBR effectively reclassified grade 2 tumors, which are often challenging to interpret using conventional grading, into biologically meaningful risk groups. These findings support the clinical utility of PDxBR as a decision-support tool for individualized risk assessment and highlight the potential of digital pathology to enhance precision oncology in breast cancer.

## Supplementary Information

Below is the link to the electronic supplementary material.

Supplementary Material 1

## Data Availability

No datasets were generated or analysed during the current study.

## Abbreviations

AI: Artificial intelligence
AUC: Area under the curve
ASCO: American Society of Clinical Oncology
CI: Confidence interval
C-index: Concordance index
ER+: Estrogen receptor-positive
ESMO: European Society for Medical Oncology
EU: European Union
H&E: Hematoxylin and eosin
HER2-: Human epidermal growth factor receptor 2-negative
HR+: Hormone receptor-positive
ID: Identifier
MINDACT: MINDACT trial variables
NA: Not applicable
NCCN: National Comprehensive Cancer Network
NPV: Negative predictive value
NGS: Nottingham Grading System
NTH: Netherlands
PDxBR: PreciseBreast
PPV: Positive predictive value
PR: Progesterone receptor
SD: Standard deviation
SE: Sensitivity
SHAP: SHapley Additive exPlanations
SP: Specificity
TRIPOD: Transparent Reporting Individual Prognosis or Diagnosis
US: United States

## References

[1] Andre F, Nofisat Ismaila;, Allison KH, Barlow WE, Collyar DE, Damodaran S et al. Biomarkers for Adjuvant Endocrine and Chemotherapy in Early-Stage Breast Cancer: ASCO Guideline Update [Internet]. 2022. Available from: www.asco.org/breast-cancer-guidelines 10.1200/JCO.22.00069 35439025

[2] Gradishar WJ, Moran MS, Abraham J, Abramson V, Aft R, Agnese D, et al. Breast cancer, version 3.2024. JNCCN J Natl Compr Cancer Netw. 2024;22:331–57. 10.6004/jnccn.2024.0035 39019058

[3] Loibl S, André F, Bachelot T, Barrios CH, Bergh J, Burstein HJ, Guidelines Committee. Early breast cancer: ESMO Clinical Practice Guideline for diagnosis, treatment and follow-up 5 behalf of the ESMO. 2024;46. Available from: 10.1016/j.annonc.2023.11.016

[4] Bray F, Laversanne M, Sung H, Ferlay J, Siegel RL, Soerjomataram I, et al. Global cancer statistics 2022: GLOBOCAN estimates of incidence and mortality worldwide for 36 cancers in 185 countries. CA Cancer J Clin. 2024;74:229–63. 38572751 10.3322/caac.21834

[5] Kim J, Harper A, McCormack V, Sung H, Houssami N, Morgan E et al. Global patterns and trends in breast cancer incidence and mortality across 185 countries. Nat Med [Internet]. 2025; Available from: http://www.ncbi.nlm.nih.gov/pubmed/39994475 10.1038/s41591-025-03502-3 39994475

[6] Siegel RL, Kratzer TB, Giaquinto AN, Sung H, Jemal A, Cancer statistics. 2025. CA Cancer J Clin [Internet]. 2025; Available from: http://www.ncbi.nlm.nih.gov/pubmed/39817679 10.3322/caac.21871 PMC11745215 39817679

[7] Pedersen RN, Esen BÖ, Mellemkjær L, Christiansen P, Ejlertsen B, Lash TL, et al. The incidence of breast cancer recurrence 10–32 years after primary diagnosis. J Natl Cancer Inst. 2022;114:391–9. 34747484 10.1093/jnci/djab202 PMC8902439

[8] Pistilli B, Lohrisch C, Sheade J, Fleming GF. Personalizing adjuvant endocrine therapy for Early-Stage hormone Receptor–Positive breast cancer. Am Soc Clin Oncol Educational Book. 2022;60–72. 10.1200/EDBK_350358 35623026

[9] Venetis K, Pescia C, Cursano G, Frascarelli C, Mane E, De Camilli E et al. The Evolving Role of Genomic Testing in Early Breast Cancer: Implications for Diagnosis, Prognosis, and Therapy. Int J Mol Sci. Multidisciplinary Digital Publishing Institute (MDPI); 2024. 10.3390/ijms25115717 PMC11172282 38891906

[10] Varga Z, Sinn P, Seidman AD. Summary of head-to-head comparisons of patient risk classifications by the 21-gene Recurrence Score^®^ (RS) assay and other genomic assays for early breast cancer. Int J Cancer. Wiley-Liss Inc.; 2019. pp. 882–93. 10.1002/ijc.32139 30653259

[11] Sparano JA, Crager MR, Gong Tang;, Gray RJ, Stemmer SM, Shak S. Development and Validation of a Tool Integrating the 21-Gene Recurrence Score and Clinical-Pathological Features to Individualize Prognosis and Prediction of Chemotherapy Benefit in Early Breast Cancer. J Clin Oncol [Internet]. 2020;39:557–64. Available from: 10.1200/JCO.20.03007 10.1200/JCO.20.03007 PMC8078482 33306425

[12] Sparano JA, Crager M, Gray RJ, Tang G, Hoag J, Baehner FL et al. Clinical and genomic risk for late breast cancer recurrence and survival. NEJM Evid. 2024;3. 10.1056/EVIDoa2300267 PMC11730053 39041867

[13] Pusztai L, Hoag JR, Albain KS, Barlow WE, Stemmer SM, Meisner A, et al. Development and validation of the RSClinN + Tool to predict prognosis and chemotherapy benefit for hormone Receptor-Positive, Node-Positive breast cancer. J Clin Oncol. 2025;43:919–28. 39621968 10.1200/JCO-24-01507 PMC11885031

[14] Zeng C, Zhang J. A narrative review of five multigenetic assays in breast cancer. Transl cancer res. AME Publishing Company; 2022. pp. 897–907. 10.21037/tcr-21-1920 PMC9091014 35571670

[15] Ji JH, Ahn SG, Yoo Y, Park SY, Kim JH, Jeong JY et al. Prediction of a Multi-Gene assay (Oncotype DX and Mammaprint) recurrence risk group using machine learning in Estrogen Receptor-Positive, HER2-Negative breast Cancer—The BRAIN study. Cancers (Basel). 2024;16. 10.3390/cancers16040774 PMC10887075 38398165

[16] Fernandez G, Zeineh J, Prastawa M, Scott R, Madduri AS, Shtabsky A, et al. Analytical validation of the precisedx digital prognostic breast cancer test in Early-Stage breast cancer. Clin Breast Cancer. 2024;24:93–e1026. 38114366 10.1016/j.clbc.2023.10.008

[17] Fernandez G, Prastawa M, Madduri AS, Scott R, Marami B, Shpalensky N et al. Development and validation of an AI-enabled digital breast cancer assay to predict early-stage breast cancer recurrence within 6 years. Breast Cancer Res. 2022;24. 10.1186/s13058-022-01592-2 PMC9764637 36539895

[18] Masud SF, Mark N, Goss T, Malinowski D, Schnitt SJ, Sparano JA et al. U.S. payer budget impact of using an AI-augmented cancer risk discrimination digital histopathology platform to identify high-risk of recurrence in women with early-stage invasive breast cancer. J Med Econ [Internet]. 2024 [cited 2025 Jul 1];27:972–81. Available from: https://www.tandfonline.com/doi/pdf/10.1080/13696998.2024.2379211 10.1080/13696998.2024.2379211 39010830

[19] Rakha EA, Tse GM, Quinn CM. An update on the pathological classification of breast cancer. Histopathology. John Wiley and Sons Inc; 2023. pp. 5–16. 10.1111/his.14786 PMC10108289 36482272

[20] Ginter PS, Idress R, D’Alfonso TM, Fineberg S, Jaffer S, Sattar AK, et al. Histologic grading of breast carcinoma: a multi-institution study of interobserver variation using virtual microscopy. Mod Pathol. 2021;34:701–9. 33077923 10.1038/s41379-020-00698-2 PMC7987728

[21] Cardoso F, van’t Veer LJ, Bogaerts J, Slaets L, Viale G, Delaloge S, et al. 70-Gene signature as an aid to treatment decisions in Early-Stage breast cancer. N Engl J Med. 2016;375:717–29. 27557300 10.1056/NEJMoa1602253

[22] Mantrala S, Ginter PS, Mitkari A, Joshi S, Prabhala H, Ramachandra V, et al. Concordance in breast cancer grading by artificial intelligence on whole slide images compares with a Multi-Institutional cohort of breast pathologists. Arch Pathol Lab Med. 2022;146:1369–77. 35271701 10.5858/arpa.2021-0299-OA

[23] Elmore JG, Longton GM, Carney PA, Geller BM, Onega T, Tosteson ANA, et al. Diagnostic concordance among pathologists interpreting breast biopsy specimens. JAMA - J Am Med Association. 2015;313:1122–32. 10.1001/jama.2015.1405 PMC4516388 25781441

[24] Filipits M, Rudas M, Jakesz R, Dubsky P, Fitzal F, Singer CF, et al. A new molecular predictor of distant recurrence in ER-positive, HER2-negative breast cancer adds independent information to conventional clinical risk factors. Clin Cancer Res. 2011;17:6012–20. 21807638 10.1158/1078-0432.CCR-11-0926

[25] Rawat RR, Ruderman D, Macklin P, Rimm DL, Agus DB. Correlating nuclear morphometric patterns with Estrogen receptor status in breast cancer pathologic specimens. NPJ Breast Cancer. 2018;4. 10.1038/s41523-018-0084-4 PMC6123433 30211313

[26] Pons L, Hernández-León L, Altaleb A, Ussene E, Iglesias R, Castillo A et al. Conventional and digital Ki67 evaluation and their correlation with molecular prognosis and morphological parameters in luminal breast cancer. Sci Rep. 2022;12. 10.1038/s41598-022-11411-5 PMC9114341 35581229

[27] Pescia C, Guerini-Rocco E, Viale G, Fusco N. Advances in early breast cancer risk profiling: from histopathology to molecular technologies. cancers (Basel). Multidisciplinary Digital Publishing Institute (MDPI); 2023. 10.3390/cancers15225430 PMC10670146 38001690

[28] Sharma A, Lövgren SK, Eriksson KL, Wang Y, Robertson S, Hartman J et al. Validation of an AI-based solution for breast cancer risk stratification using routine digital histopathology images. Breast Cancer Research [Internet]. 2024 [cited 2025 Jul 9];26:1–10. Available from: https://breast-cancer-research.biomedcentral.com/articles/10.1186/s13058-024-01879-6 10.1186/s13058-024-01879-6 PMC11323658 39143539

[29] Witowski J, Zeng KG, Cappadona J, Elayoubi J, Choucair K, Chiru ED et al. Multi-modal AI for comprehensive breast cancer prognostication. 2024 [cited 2025 Jul 9]; Available from: https://arxiv.org/pdf/2410.21256 10.1038/s41467-026-73088-y PMC13338129 42161927

