# Supplementary material for: External validation of precisebreast, a digital prognostic test for predicting breast cancer recurrence, in an early-stage cohort from the Netherlands
Source: Breast Cancer Res. 2025 Aug 20;27:152. doi: 10.1186/s13058-025-02104-8 (PMC12369158; doi:10.1186/s13058-025-02104-8)

**External validation of PreciseBreast, a digital prognostic test for predicting breast cancer recurrence, in an early-stage cohort from the Netherlands**

Pieter J. Westenend, Claudia Meurs, Gerardo Fernandez, Marcel Prastawa,

Abishek Sainath Madduri, Aaron Feliz, Juan Carlos Mejias, Alex Shtabsky, Xiaozhu Zhang, Brandon Veremis, Rebecca DeAngel, Michael J. Donovan

**Supplemental Material**

Figure S1. SHAP plot of the PDxBR image feature AI-grade model

Table S1. Cox proportional hazards and C-index analysis on imaging features in the PDxBR

Table S2. Event types stratified by risk group and assay type

Figure S2. Kaplan-Meier survival curve for PDxBR predicting distant metastasis in the NTH cohort

Table S3. Performance Matrix for Distant Metastasis only

Figure S3. AUC comparing histologic grade vs AI-grade for 739 patients over 5-years

Table S4. Histologic grade vs PDxBR AI-grade redistribution

Figure S4. Kaplan-Meier survival curves comparing histology grade vs AI-grade risk

Figure S5. Kaplan-Meier survival curve for PDxBR using histology grade 2 patients only

Table S5. Demographic characteristics for patients with MammaPrint result

Table S6. Performance metrics in patients with MammaPrint result

Table S7. Event type in the patients with MammaPrint Risk result

Table S8. Event analysis in 252 patients with MammaPrint results

Figure S6. Kaplan-Meier survival curve of MammaPrint risk stratification

**Figure S1. SHAP plot of the PDxBR image feature AI-grade model**


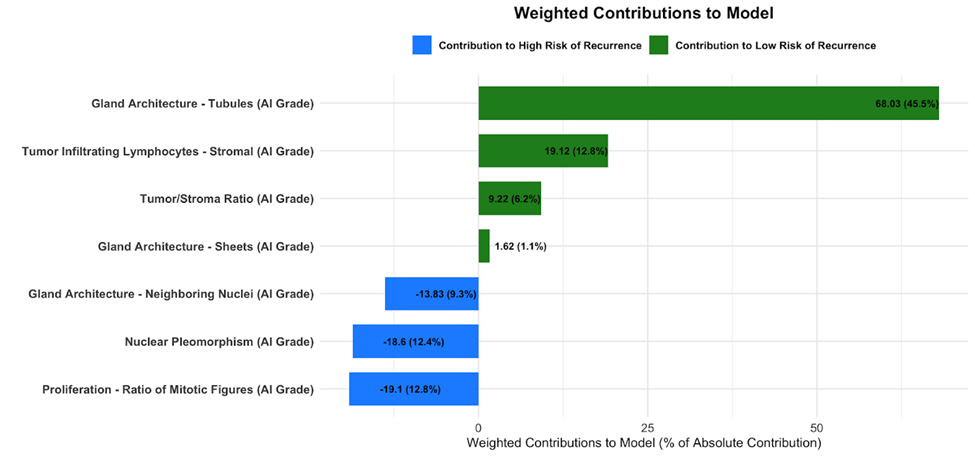


SHAP plot detailing differential weighting (importance) of individual morphological features.

AI, artificial intelligence.

**Table S1. Cox proportional hazards and C-index analysis on imaging features in the PDxBR model**

|  | **Feature** | **HR** | **95% CI lower** | **95% CI upper** | **P value** | **C index** |
| --- | --- | --- | --- | --- | --- | --- |
| Gland Architecture - Sheets (AI Grade) | tumor4_Tubules_x1minus_Sheets | 1.00000e-03 | 0.000000 | 0.016 | 1.86e-06 | 0.364 |
| Gland Architecture - Tubules (AI Grade) | tumor4_Sheetsx_x1minus_Tubulesx | 1.03210e+01 | 3.798000 | 28.048 | 4.74e-06 | 0.626 |
| Nuclear Pleomorphism (AI Grade) | NucleiSizeHighByAveInvEpi | 1.88100 | 9.520000e-01 | 3.720 | 6.92e-02 | 0.541 |
| Proliferation - Ratio of Mitotic Figures (AI Grade) | MitoNucleiRatioInvEpi | 1.18371e+204 | 3.830409e+43 | Infinity | 1.27e-02 | 0.572 |

**Table S2. Event types* stratified by risk group and assay type**

|  | **Death** | **Last known follow-up** | **Metastasis** | **Recurrence**** |  | **Subtotal (n)** |
| --- | --- | --- | --- | --- | --- | --- |
| **Actual Events, n** | 81 | 609 | 25 | 24 |  | 130 |
| **Risk Group, n (%)** |  |  |  |  |  |  |
| **PDxBR** |  |  |  |  |  |  |
| Low-risk | 20 (24.7) | 361 (59.3) | 9 (36.0) | 10 (41.7) |  | 400 |
| High-risk | 61 (75.3) | 248 (40.7) | 16 (64.0) | 14 (58.3) |  | 339 |
| **PDxBR AI-grade** |  |  |  |  |  |  |
| Low-risk | 23 (28.4) | 334 (54.8) | 4 (16.0) | 11 (45.8) |  | 372 |
| High-risk | 58 (71.6) | 275 (45.2) | 21 (84.0) | 13 (54.2) |  | 367 |
| **PDxBR Clinical** |  |  |  |  |  |  |
| Low-risk | 31 (38.3) | 427 (70.1) | 11 (44.0) | 10 (41.7) |  | 479 |
| High-risk | 50 (61.7) | 182 (29.9) | 14 (56.0) | 14 (58.3) |  | 260 |
| **MINDACT** |  |  |  |  |  |  |
| Low-risk | 43 (53.1) | 407 (66.8) | 8 (32.0) | 9 (37.5) |  | 467 |
| High-risk | 38 (46.9) | 202 (33.2) | 17 (68.0) | 15 (62.5) |  | 272 |
| **MammaPrint (MP) Subgroup Analysis** |  |  |  |  |  |  |
| **Actual Events, n** | 7 | 236 | 5 | 4 |  | 16 |
| **MammaPrint Risk Group, n (%)** |  |  |  |  |  |  |
| Low-risk | 4 (57.1) | 156 (66.1) | 2 (40.0) | 4 (100.0) |  | 166 |
| High-risk | 3 (42.9) | 80 (33.9) | 3 (60.0) | 0 (0.0) |  | 86 |

#### AI, artificial intelligence; PDxBR, PreciseBreast.

*Any recurrence

**Second primary or local/regional

**Figure S2. Kaplan-Meier curve for PDxBR predicting distant metastasis in the NTH cohort**

**
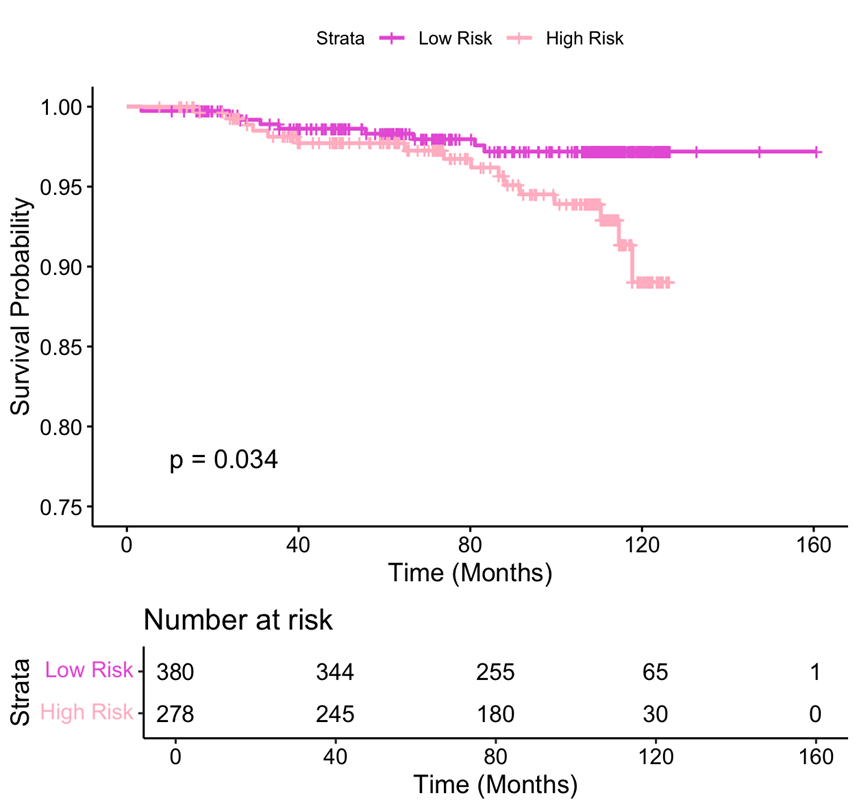
**

#### **Table S3. Performance Matrix for Distant Metastasis only**

|  | **C Index**  **(95% CI)** | **HR**  **(95% CI)** | **SE**  **(95% CI)** | **SP**  **(95% CI)** | **PPV**  **(95% CI)** | **NPV**  **(95% CI)** |
| --- | --- | --- | --- | --- | --- | --- |
| **MammaPrint** | 0.64  (0.40-0.89) | 3.18  (0.53-19.08) | 0.60  (0.21-0.91) | 0.66  (0.60-0.72) | 0.03  (0.01-0.10) | 0.99  (0.96-1.0) |
| **PDxBR** | 0.69  (0.60-0.78) | 2.36  (1.04-5.35) | 0.64  (0.44-0.80) | 0.55  (0.51-0.58) | 0.05  (0.03-0.08) | 0.98  (0.96-0.99) |
| **PDxBR AI-grade** | 0.71  (0.62-0.80) | 6.35  (2.17-18.53) | 0.84  (0.65-0.94) | 0.52  (0.48-0.55) | 0.06  (0.04-0.09) | 0.99  (0.97-1.00) |
| **PDxBR Clinical** | 0.65  (0.54-0.76) | 2.52  (1.15-5.56) | 0.56  (0.37-0.74) | 0.66  (0.62-0.69) | 0.05  (0.03-0.09) | 0.98  (0.96-0.99) |
| **MINDACT** | 0.68  (0.59-0.77) | 3.88  (1.67-8.98) | 0.68  (0.48-0.83) | 0.64  (0.61-0.68) | 0.06  (0.04-0.10) | 0.98  (0.97-0.99) |

**Figure S3. AUC comparing histologic grade vs AI-grade for 739 patients over 5-years**

**
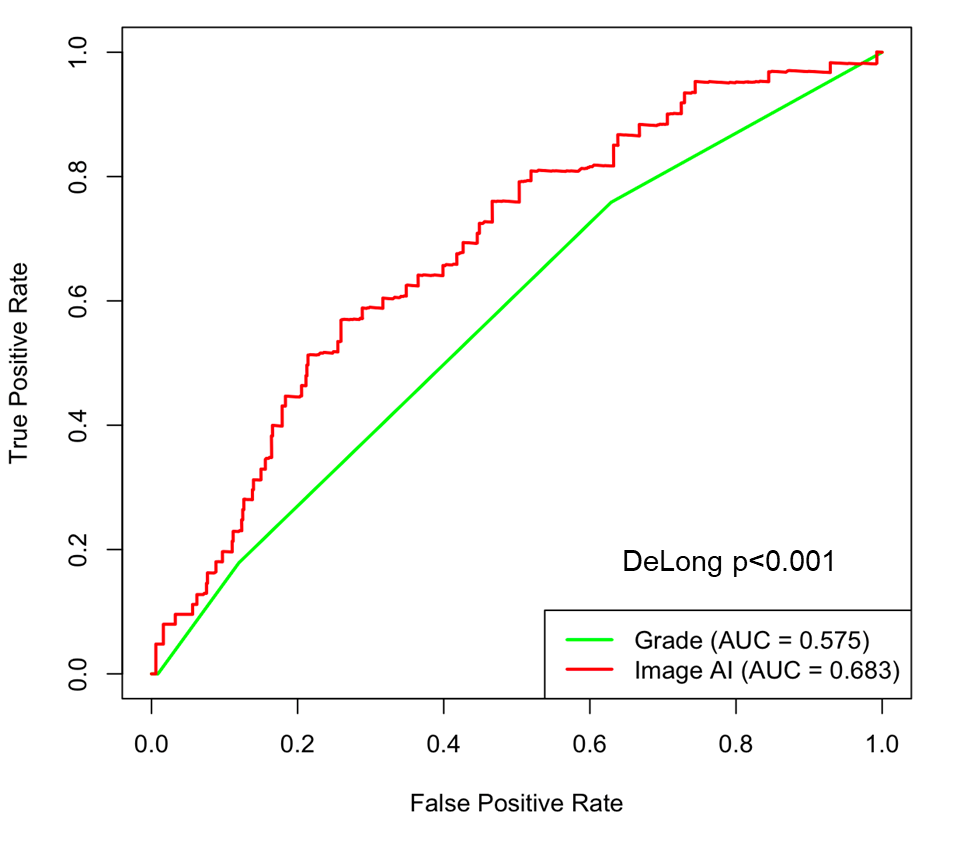
**

**Table S4. Histologic grade vs PDxBR AI-grade redistribution**

| **Histologic**  **Grade** | **PDxBR AI-grade**  **Low-risk, N (%)** | **PDxBR AI-grade**  **High-risk, N (%)** | **Total** |
| --- | --- | --- | --- |
| 1 | 203 (76.3) | 63 (23.7) | 266 |
| 2 | 149 (39.1) | 232 (60.9) | 381 |
| 3 | 16 (18.6) | 70 (81.4) | 86 |

PDxBR, PreciseBreast; 6 patients were missing an assigned histologic grade

**Figure S4. Kaplan-Meier survival curves comparing histology grade vs AI-grade risk**


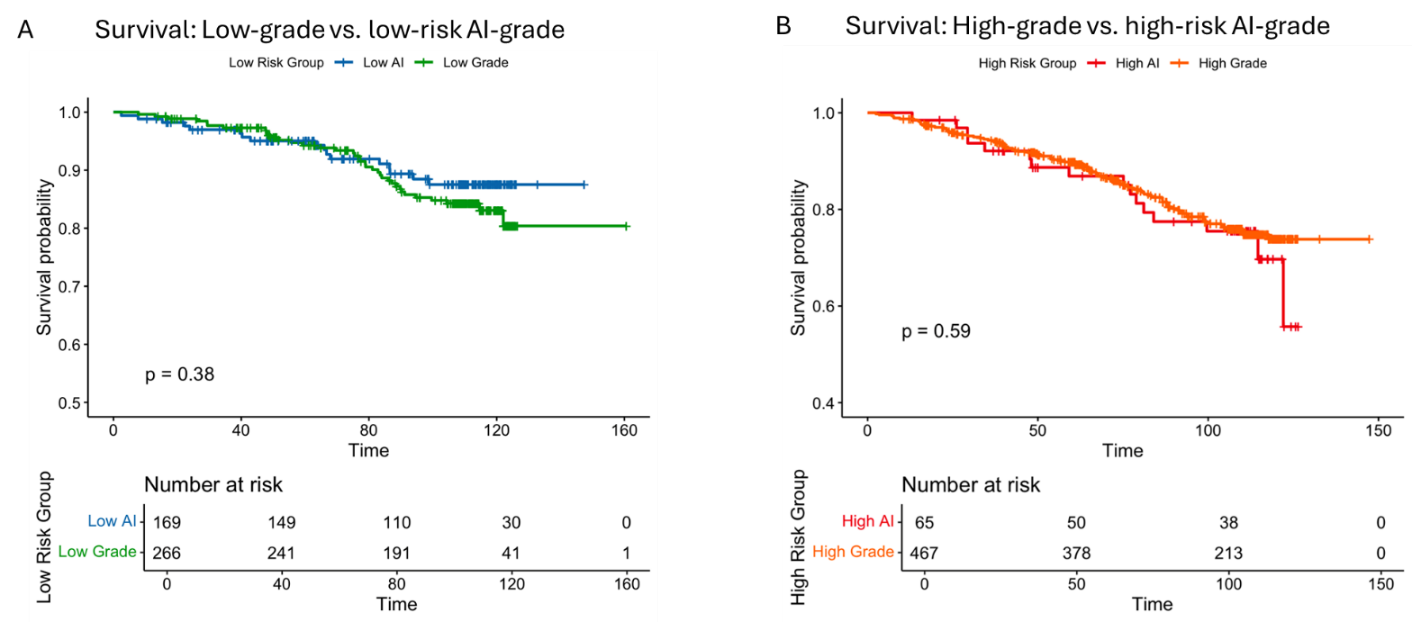


A) KM curve comparing histology low-grade vs. AI-grade low-risk; B) KM curve comparing histology high-grade (NGS 2+3) vs. AI-grade high-risk

**Figure S5. Kaplan-Meier survival curve for PDxBR using histology grade 2 patients only**


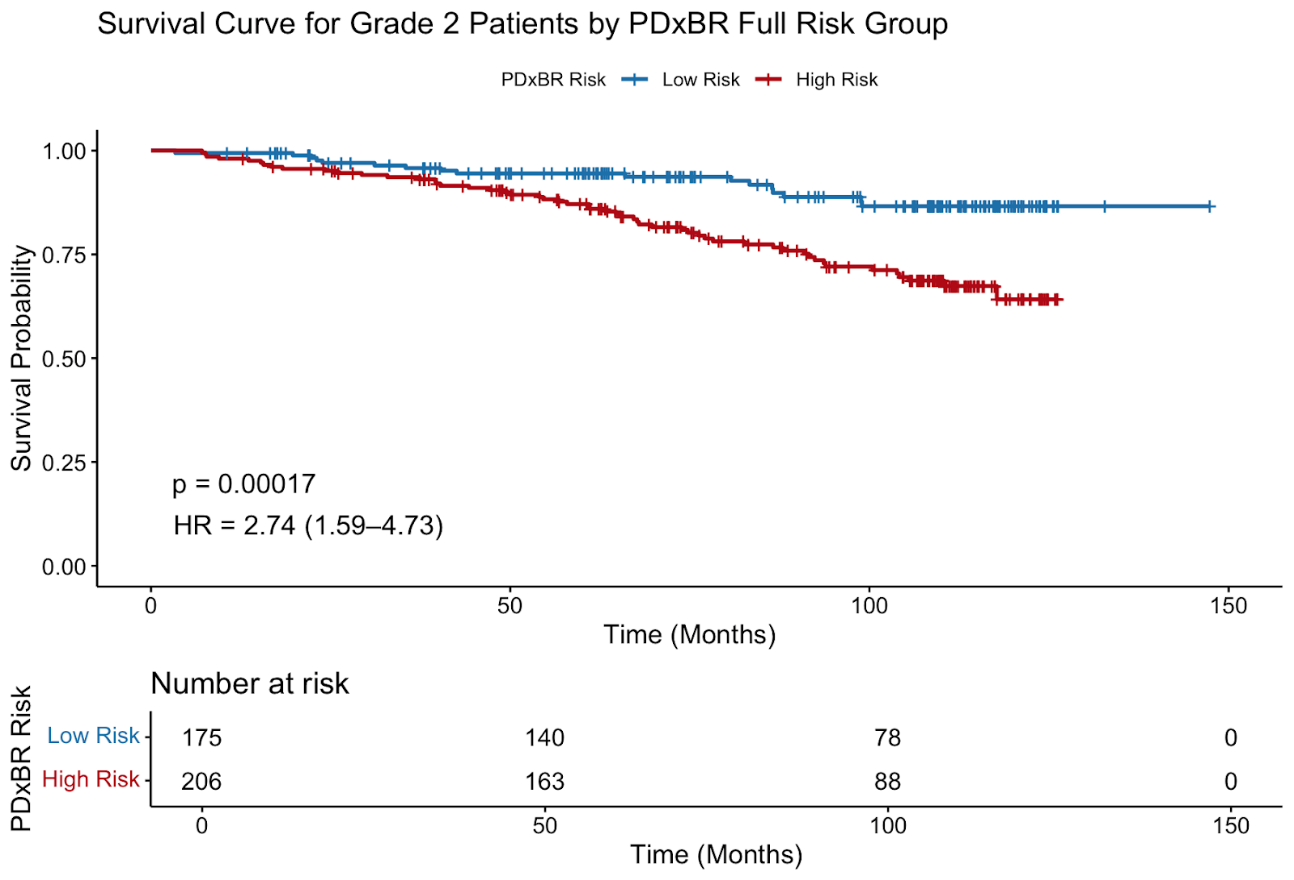


### **Table S5. Demographic characteristics for patients with MammaPrint result**

| **Demographic Characteristics** | **N = 252** |
| --- | --- |
| **Age at Diagnosis, years, median (Min, Max)** | 57 (29, 72) |
| ≤50, n (%) | 67 (26.6) |
| >50, n (%) | 185 (73.4) |
| **Tumor Size, n (%)** |  |
| ≤2.5 cm | 230 (91.3) |
| >2.5 cm | 21 (8.3) |
| >5 cm | 1 (0.4) |
| **Anatomic Stage, n (%)** |  |
| I | 159 (63.1) |
| II | 92 (36.5) |
| III | 1 (0.4) |
| IV | 0 (0) |
| **Lymph Node, n (%)** |  |
| Negative | 211 (83.7) |
| Positive 1-3 | 41 (16.3) |
| **Tumor Grade** |  |
| 1 | 65 (25.8) |
| 2 | 168 (66.7) |
| 3 | 19 (7.5) |
| **ER+, n (%)** | 252 (100) |
| **PR+, n (%)** | 229 (92.9) |
| **HER-, n (%)** | 252 (100) |
| **Treatments Received, n (%)** |  |
| Chemotherapy | 75 (29.8) |
| Endocrine | 216 (85.7) |
| Radiation | 193 (76.6) |
| **Total events, n (%)** | 16 (6.4) |
| **Follow up time, months, mean (SD)** | 59.1 (23.5) |
| **MammaPrint** |  |
| Low-risk | 86 (34.1) |
| High-risk | 166 (65.9) |
| **PDxBR** |  |
| Low-risk | 145 (57.5) |
| High-risk | 107 (42.5) |
| **PDxBR AI** |  |
| Low-risk | 116 (46.0) |
| High-risk | 136 (54.0) |
| **PDxBR Clinical** |  |
| Low-risk | 187 (74.2) |
| High-risk | 65 (25.8) |

ER, estrogen receptor; PR, progesterone receptor; HER, human epidermal growth factor receptor; AI, artificial intelligence; PDxBR, PreciseBreast.

**Table S6. Performance metrics in patients with MammaPrint (MP) result**

|  | **C Index**  **(95% CI)** | **HR**  **(95% CI)** | **SE**  **(95% CI)** | **SP**  **(95% CI)** | **PPV**  **(95% CI)** | **NPV**  **(95% CI)** |
| --- | --- | --- | --- | --- | --- | --- |
| **MammaPrint** | 0.52  (0.39-0.65) | 1.19  (0.43-3.29) | 0.38  (0.18-0.62) | 0.66  (0.6-0.72) | 0.07  (0.03-0.15) | 0.94  (0.89-0.97) |
| **PDxBR** | 0.51  (0.41-0.61) | 0.55  (0.19-1.58) | 0.31  (0.13-0.56) | 0.57  (0.5-0.63) | 0.05  (0.02-0.11) | 0.92  (0.87-0.96) |
| **PDxBR AI-grade** | 0.51  (0.38-0.63) | 1.28  (0.47-3.54) | 0.62  (0.38-0.82) | 0.47  (0.4-0.53) | 0.07  (0.04-0.13) | 0.95  (0.89-0.98) |
| **PDxBR Clinical** | 0.58  (0.46-0.71) | 0.82  (0.23-2.88) | 0.19  (0.06-0.44) | 0.74  (0.68-0.79) | 0.05  (0.01-0.13) | 0.93  (0.88-0.96) |
| **MINDACT** | 0.55  (0.42-0.68) | 1.32  (0.46-3.83) | 0.31  (0.13-0.56) | 0.69  (0.63-0.75) | 0.06  (0.03-0.14) | 0.94  (0.89-0.97) |

AI, artificial intelligence; PDxBR, PreciseBreast.

#### **Table S7. Event type in the patients with MammaPrint Risk result**

|  | **Death** | **Metastasis** | **Recurrence** | **Subtotal** |
| --- | --- | --- | --- | --- |
| **Actual Events, n** | 7 | 5 | 4 | 16 |
| Age ≤ 50, n (%) | 0 (0.0) | 1 (20.0) | 1 (25.0) | 2 |
| Age > 50, n (%) | 7 (100.0) | 4 (80.0) | 3 (75.0) | 14 |
| **Tumor Size, n (%)** |  |  |  |  |
| ≤ 2.5 cm | 7 (100.0) | 4 (80.0) | 4 (100.0) | 15 |
| > 2.5 cm | 0 (0.0) | 1 (20.0) | 0 (0.0) | 1 |
| **Tumor Grade** |  |  |  |  |
| 1 | 6 (85.7) | 5 (100.0) | 4 (100.0) | 15 |
| >1 | 1 (14.3) | 0 (0.0) | 0 (0.0) | 1 |
| **MammaPrint Risk Group, n (%)** |  |  |  |  |
| Low-risk | 4 (57.1) | 2 (40.0) | 4 (100.0) | 10 |
| High-risk | 3 (42.9) | 3 (60.0) | 0 (0.0) | 6 |
| **Lymph Node, n (%)** |  |  |  |  |
| Negative | 6 (85.7) | 5 (100.0) | 3 (75.0) | 14 |
| Positive | 1 (14.3) | 0 (0.0) | 1 (25.0) | 2 |
| **Median Follow up time (month) = 45 months** |  |  |  |  |

**Table S8. Event analysis in 252 patients with MammaPrint results**

|  | **Death** | **Last known**  **follow-up** | **Metastasis** | **Recurrence** | **Subtotal (n)** |
| --- | --- | --- | --- | --- | --- |
| **Actual Events, n** | 7 | 236 | 5 | 4 | 16 |
| **Risk Group, n (%)** |  |  |  |  |  |
| **PDxBR** |  |  |  |  |  |
| Low-risk | 5 (71.4) | 134 (56.8) | 3 (60.0) | 3 (75.0) | 145 |
| High-risk | 2 (28.6) | 102 (43.2) | 2 (40.0) | 1 (25.0) | 107 |
| **PDxBR AI-grade** |  |  |  |  |  |
| Low-risk | 2 (28.6) | 110 (46.6) | 2 (40.0) | 2 (50.0) | 116 |
| High-risk | 5 (71.4) | 126 (53.4) | 3 (60.0) | 2 (50.0) | 136 |
| **PDxBR Clinical** |  |  |  |  |  |
| Low-risk | 6 (85.7) | 174 (73.7) | 4 (80.0) | 3 (75.0) | 187 |
| High-risk | 1 (14.3) | 62 (26.3) | 1 (20.0) | 1 (25.0) | 65 |
| **MINDACT** |  |  |  |  |  |
| Low-risk | 5 (71.4) | 164 (69.5) | 3 (60.0) | 3 (75.0) | 175 |
| High-risk | 2 (28.6) | 72 (30.5) | 2 (40.0) | 1 (25.0) | 77 |
| **MammaPrint** |  |  |  |  |  |
| Low-risk | 4 (57.1) | 156 (66.1) | 2 (40.0) | 4 (100.0) | 166 |
| High-risk | 3 (42.9) | 80 (33.9) | 3 (60.0) | 0 (0.0) | 86 |

**Figure S6. Kaplan-Meier survival curve of MammaPrint risk stratification**


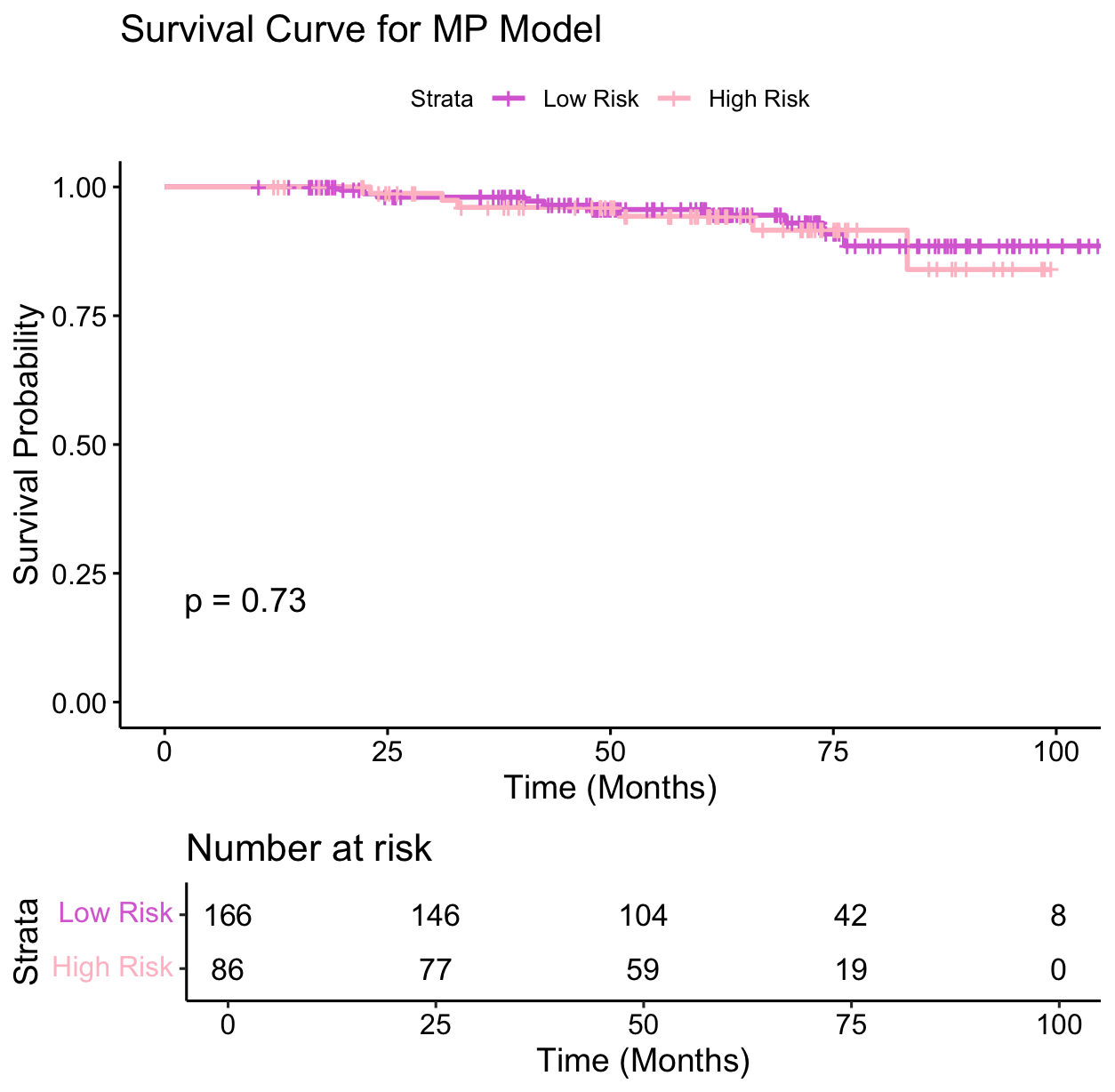

Supplement: Supplementary file 1 — Supplementary Material 1 [file 13058_2025_2104_MOESM1_ESM.docx]
